# Plant-Derived Bioactive Metabolites from the Sonoran Desert: Redox Regulation, Nrf2/NF-κB Signaling, and Emerging Therapeutic Applications

**DOI:** 10.3390/ijms27104634

**Published:** 2026-05-21

**Authors:** Lidianys Maria Lewis-Luján, Annette Pulcherie Iloki-Lewis, Diego Emmanuel Guerrero-Magaña, Mikhail A. Osadchuk, Maxim V. Trushin, Juan Carlos Galvez-Ruiz, Judas Tadeo Vargas Durazo, Cinthia Jhovanna Perez-Martinez, Maria Guadalupe Burboa-Zazueta, Ana V. Torres-Figueroa, Sergio Trujillo Lopez, Simon Bernard Iloki-Assanga

**Affiliations:** 1 Department of Biological Chemical Sciences, University of Sonora, Blvd. Luis Encinas, Col. Centro, Hermosillo 83000, Sonora, Mexico; lidianys.lewis@unison.mx (L.M.L.-L.); diego.guerrero@unison.mx (D.E.G.-M.); juan.galvez@unison.mx (J.C.G.-R.); judas.vargas@unison.mx (J.T.V.D.); jhovanna.perez@unison.mx (C.J.P.-M.); anavaleria.torres@unison.mx (A.V.T.-F.); 2 Department of Medicine and Health Sciences, University of Sonora, Blvd. Luis Encinas, Col. Centro, Hermosillo 83000, Sonora, Mexico; ilokiannette@gmail.com (A.P.I.-L.); sergio.trujillo@unison.mx (S.T.L.); 3 Department of Polyclinic Therapy, Sechenov First Moscow State Medical University, 119435 Moscow, Russia; osadchuk.mikhail@yandex.ru; 4 Institute of Fundamental Medicine and Biology, Kazan Federal University, 420008 Kazan, Russia; mtrushin@mail.ru; 5 Department of Scientific and Technological Research, University of Sonora, Luis Encinas y Rosales, Centro, Hermosillo 83000, Sonora, Mexico; maria.burboa@unison.mx

**Keywords:** Sonoran desert plants, plant-derived metabolites, oxidative stress, Nrf2/NF-κB, redox signaling, inflammation, phytochemicals

## Abstract

Plant-derived bioactive metabolites have emerged as promising modulators of oxidative stress and inflammation, two interconnected processes involved in the pathogenesis of numerous chronic diseases. Arid ecosystems, particularly the Sonoran Desert, constitute an underexplored source of structurally diverse phytochemicals with significant pharmacological potential. This review provides a comprehensive overview of major classes of plant-derived bioactives, including polyphenols, flavonoids, terpenoids, and alkaloids, with emphasis on their molecular mechanisms of antioxidant and anti-inflammatory action. These compounds exert cytoprotective effects through direct reactive oxygen species (ROS) scavenging and indirect regulation of endogenous defense systems, primarily via activation of the Nrf2/Keap1 pathway and suppression of NF-κB signaling. Additional pathways, including MAPK, PI3K/Akt, AMPK, and mitochondrial regulatory networks, are discussed as critical mediators of redox balance and inflammatory control. Particular attention is given to Sonoran Desert plant species such as *Bucida buceras*, *Phoradendron californicum*, *Larrea tridentata*, *Opuntia* spp., and *Agave deserti*, all of which demonstrate promising biological activities associated with enhanced adaptation to environmental stress. Experimental approaches used to evaluate phytochemical bioactivity, including chemical assays, cellular models, omics technologies, and translational strategies, are also examined. Furthermore, this review discusses current limitations related to bioavailability, phytochemical variability, and clinical validation, highlighting emerging nanodelivery systems and precision medicine approaches as potential solutions. Collectively, the evidence supports the therapeutic relevance of Sonoran Desert plant bioactives as multi-target agents for modulating oxidative stress, inflammation, and chronic disease progression.

## 1. Introduction

Oxidative stress results from an imbalance between the production of ROS and the capacity of antioxidant defense systems to neutralize them. Excessive ROS accumulation leads to lipid peroxidation, protein oxidation, and DNA damage, contributing to the pathogenesis of numerous chronic diseases [1]. Importantly, oxidative stress is closely linked to chronic inflammation, forming a self-amplifying cycle that exacerbates tissue injury and disease progression [2].

At the molecular level, oxidative stress activates key signaling pathways such as nuclear factor kappa B (NF-κB), which regulates pro-inflammatory cytokine expression, whereas antioxidant responses are primarily mediated by the nuclear factor erythroid 2-related factor 2 (Nrf2) pathway [3]. The interplay between these pathways is critical for maintaining cellular homeostasis.

Plant-derived bioactive compounds, particularly polyphenols and flavonoids, have attracted significant attention due to their ability to modulate both oxidative stress and inflammation. These compounds act through direct antioxidant mechanisms as well as through the regulation of intracellular signaling pathways [4].

Plants from arid and semi-arid environments represent a unique and underexplored source of bioactive metabolites. Environmental stress conditions, such as drought, high temperature, and ultraviolet radiation, stimulate the production of secondary metabolites with enhanced biological activity [5]. In this context, the Sonoran Desert harbors plant species with significant therapeutic potential, including *Bucida buceras*, *Phoradendron californicum*, *Larrea tridentata*, *Opuntia* spp., and *Agave deserti* [6,7,8].

**Figure 1:**
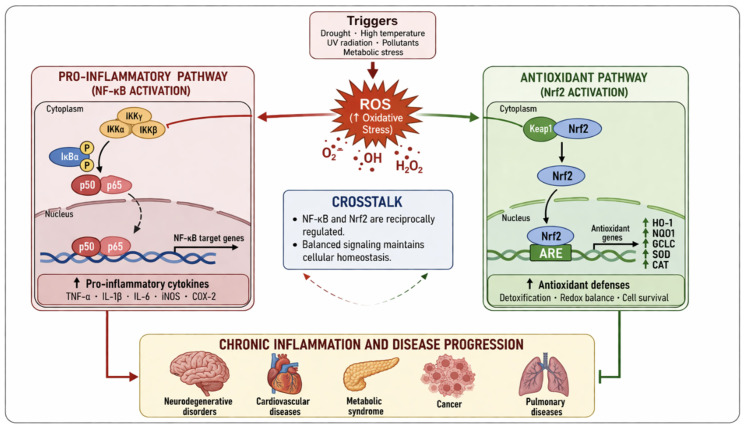
Interplay between oxidative stress and inflammation.

This review aims to provide a comprehensive and critical overview of plant-derived bioactive metabolites, focusing on their molecular mechanisms of antioxidant and anti-inflammatory activity, experimental validation, and therapeutic potential. The reciprocal interaction between oxidative stress and inflammatory signaling pathways is summarized in Figure 1, highlighting the central crosstalk between ROS production, NF-κB activation, and Nrf2-mediated antioxidant defense mechanisms.

Excessive ROS activates the NF-κB pathway, promoting the expression of pro-inflammatory cytokines (TNF-α, IL-6, IL-1β) [1,2,3]. Conversely, activation of the Nrf2 pathway induces antioxidant and cytoprotective gene expression through antioxidant response elements (AREs). Crosstalk between these pathways regulates cellular homeostasis and contributes to chronic disease progression.

Unlike previous generalized phytochemical reviews, this work specifically integrates Sonoran Desert-derived metabolites within a mechanistic redox–inflammation framework, emphasizing molecular signaling convergence and translational potential.

## 2. Methods

**Figure 2:**
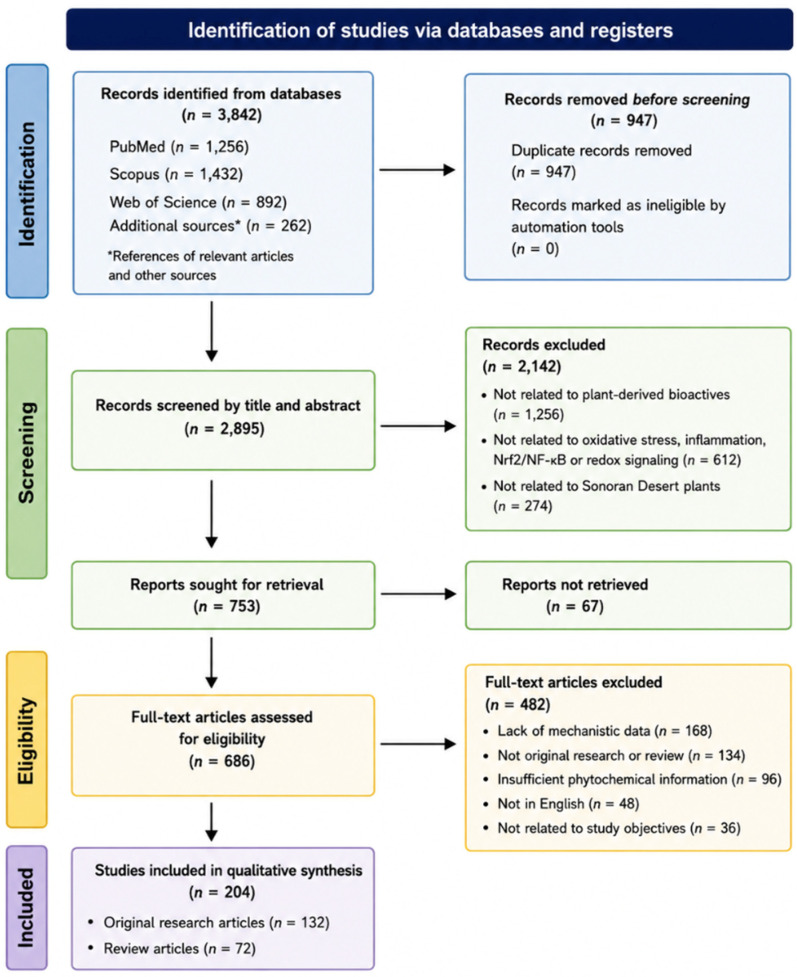
Flow diagram showing the literature selection and screening for this narrative review. The search covered the period from January 2015 to March 2025.

This narrative, non-systematic review was conducted using a structured and transparent literature search strategy to ensure scientific rigor, reproducibility, and methodological consistency. Elements commonly used in PRISMA-style reporting were incorporated to describe the literature screening and selection process. A flow diagram summarizing the study identification and selection process is presented in Figure 2.

A comprehensive literature search was conducted using PubMed, Scopus, and Web of Science databases to identify relevant studies published between January 2015 and March 2025. Particular emphasis was placed on recent high-impact publications (2020–2025) addressing oxidative stress, inflammation, redox signaling, phytochemical bioactivity, and Sonoran Desert-derived metabolites. The search strategy combined controlled keywords and Boolean operators, including “oxidative stress”, “plant-derived bioactives”, “phytochemicals”, “polyphenols”, “flavonoids”, “Nrf2”, “NF-κB”, “redox signaling”, “anti-inflammatory mechanisms”, and “Sonoran Desert plants”. Studies were selected according to the following inclusion criteria:

- Peer-reviewed original research articles and review papers.
- Studies investigating antioxidant and/or anti-inflammatory mechanisms of plant-derived bioactive compounds.
- Articles focused on molecular pathways, including Nrf2/Keap1, NF-κB, MAPK, PI3K/Akt, AMPK, and mitochondrial regulation.
- Experimental studies using chemical assays, cellular models, or in vivo systems.
- Articles published in English.

The exclusion criteria included:

- Non-peer-reviewed articles, editorials, conference abstracts, and duplicate records.
- Studies lacking mechanistic or molecular relevance.
- Publications with insufficient methodological detail or unclear biological outcomes.
- Studies unrelated to the objectives of oxidative stress, inflammation, redox signaling, or Sonoran Desert-derived phytochemicals.

A summary of the literature screening and study selection process is presented in Figure 2. In total, 204 studies meeting the inclusion criteria were included in the final qualitative synthesis. A critical approach was adopted to evaluate the strengths and limitations of the current evidence. Data extraction focused on the following parameters:

- Phytochemical class (polyphenols, flavonoids, terpenoids, alkaloids, polysaccharides).
- Experimental model (chemical assays, cell lines, animal models, omics approaches).
- Molecular mechanisms of action.
- Signaling pathways involved.
- Antioxidant and anti-inflammatory biological effects.
- Translational and therapeutic relevance.

The selected studies were qualitatively analyzed and categorized according to mechanistic relevance, phytochemical composition, experimental evidence, and therapeutic potential. Particular emphasis was placed on Sonoran Desert plant species and their integration into redox and inflammatory signaling pathways.

## 3. Bioactive Metabolites and Molecular Diversity

**Figure 3:**
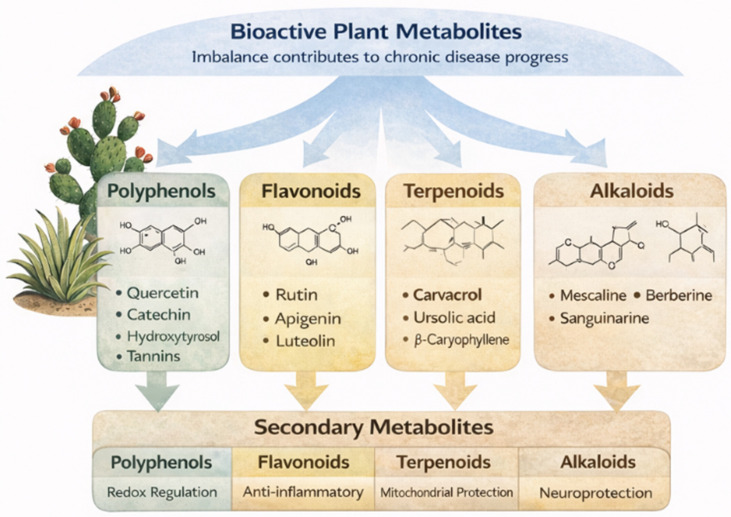
Major classes of plant-derived bioactive metabolites, including polyphenols, flavonoids, terpenoids, and alkaloids, highlighting their structural diversity and biological activities [9,10,11,12].

The principal categories of plant-derived bioactive metabolites, including polyphenols, flavonoids, terpenoids, and alkaloids, together with their representative compounds and biological functions, are summarized in Figure 3. Plant-derived bioactive metabolites represent a structurally diverse and functionally dynamic class of secondary metabolites, including polyphenols, flavonoids, terpenoids, and alkaloids.

These compounds play a crucial role in plant adaptation to environmental stress and exhibit significant pharmacological potential in human health. Their biological activity is largely determined by their chemical structure, redox properties, and capacity to interact with multiple molecular targets, including transcription factors, enzymes, and signaling cascades [9,10].

In particular, plants from arid and semi-arid environments synthesize elevated levels of bioactive metabolites as adaptive responses to extreme conditions such as high ultraviolet radiation, drought, and temperature fluctuations.

These stress-induced metabolic adaptations result in the accumulation of compounds with enhanced antioxidant and anti-inflammatory properties [11]. Importantly, these metabolites exhibit multi-target mechanisms, allowing them to simultaneously modulate oxidative stress, inflammation, mitochondrial function, and cellular signaling pathways, which is particularly relevant in complex diseases such as neurodegeneration and metabolic disorders [12].

### 3.1. Polyphenols

Polyphenols constitute one of the most abundant and biologically active classes of plant secondary metabolites. Structurally, they are characterized by multiple hydroxyl groups attached to aromatic rings, which confer strong redox properties and enable efficient scavenging of ROS. These compounds neutralize free radicals primarily through hydrogen atom transfer (HAT) and single-electron transfer (SET) mechanisms, thereby interrupting oxidative chain reactions such as lipid peroxidation [13,14].

Importantly, Sonoran Desert species such as Larrea tridentata accumulate high levels of phenolic compounds and lignans, particularly nordihydroguaiaretic acid (NDGA), as adaptive responses to intense ultraviolet radiation and drought stress. These metabolites exhibit strong antioxidant and anti-inflammatory activities associated with ROS scavenging and modulation of Nrf2/NF-κB signaling pathways.

Beyond direct antioxidant activity, polyphenols exert profound regulatory effects on intracellular signaling pathways. A key mechanism involves the activation of the Nrf2/Keap1 pathway, which promotes the transcription of antioxidant enzymes such as heme oxygenase-1 (HO-1), NAD(P)H quinone oxidoreductase 1 (NQO1), and glutathione-related enzymes. Concurrently, polyphenols inhibit the activation of NF-κB, reducing the expression of pro-inflammatory mediators including TNF-α, IL-6, and IL-1β [15,16].

Recent studies have demonstrated that polyphenols function as mild electrophiles, triggering adaptive cellular responses rather than acting solely as direct antioxidants. This hormetic effect enhances endogenous antioxidant defenses and contributes to long-term cytoprotection [17]. Additionally, structure-activity relationships indicate that the number and position of hydroxyl groups, degree of conjugation, and molecular size critically influence their antioxidant efficiency and bioavailability [18].

### 3.2. Flavonoids

Flavonoids, a subclass of polyphenols, include compounds such as quercetin, kaempferol, and catechins, and are widely recognized for their potent antioxidant and anti-inflammatory properties. These compounds exhibit ROS-scavenging activity through electron donation and metal chelation, particularly targeting transition metals such as Fe^2+^ and Cu^2+^, thereby inhibiting Fenton-type reactions and reducing hydroxyl radical formation [19,20].

At the molecular level, flavonoids exert anti-inflammatory effects primarily through inhibition of the NF-κB signaling pathway, achieved by preventing the phosphorylation and degradation of IκBα. Additionally, flavonoids modulate MAPK pathways (ERK, JNK, and p38), which are involved in cellular stress responses and cytokine production [21]. Flavonoids identified in *Opuntia ficus-indica* and other Sonoran cacti include quercetin derivatives, kaempferol glycosides, and betalain-associated phenolics, which contribute to protection against oxidative stress induced by extreme arid environmental conditions.

Emerging evidence also indicates that flavonoids regulate additional signaling networks, including PI3K/Akt and AMPK pathways, contributing to improved cellular survival, metabolic regulation, and mitochondrial function [22]. Importantly, flavonoids demonstrate significant neuroprotective and cardioprotective effects, highlighting their relevance in chronic disease prevention [22,23].

Their biological activity is strongly influenced by structural features, including hydroxylation patterns, glycosylation, and the presence of double bonds. These factors determine their solubility, absorption, metabolism, and overall bioavailability [24].

### 3.3. Terpenoids

Terpenoids, also known as isoprenoids, represent one of the largest classes of natural products and include compounds such as carotenoids, monoterpenes, diterpenes, and triterpenes. These molecules play a critical role in cellular protection by stabilizing biological membranes and preventing lipid peroxidation [25].

Carotenoids, for example, are highly effective at quenching singlet oxygen and scavenging peroxyl radicals due to their conjugated double-bond systems. This ability allows them to reduce oxidative damage in lipid-rich environments such as cellular membranes [26].

In addition to their antioxidant effects, terpenoids modulate key signaling pathways involved in inflammation and cell survival. These include the PI3K/Akt pathway, which promotes cell survival, and inhibition of NF-κB signaling, thereby reducing inflammatory responses. Some terpenoids also regulate apoptotic pathways and mitochondrial function, contributing to cytoprotection [27].

Several Sonoran Desert plants also produce terpenoid-rich extracts as adaptive mechanisms against abiotic stress. In species such as *Agave deserti*, terpenoids and related secondary metabolites may contribute to membrane stabilization and oxidative stress tolerance under prolonged drought and high-temperature exposure.

Recent studies have highlighted the role of terpenoids in regulating metabolic and immune responses, suggesting their potential in the treatment of metabolic disorders and inflammatory diseases [28]. Structural variations such as chain length, degree of unsaturation, and functional group modifications significantly influence their biological activity and pharmacokinetic properties.

### 3.4. Alkaloids

Alkaloids are nitrogen-containing secondary metabolites with diverse chemical structures and significant pharmacological activities. These compounds interact with a wide range of biological targets, including enzymes, receptors, and ion channels, making them highly versatile bioactive agents [29].

Among alkaloids, berberine has been extensively studied for its antioxidant and anti-inflammatory properties. Its mechanism of action involves activation of the AMPK signaling pathway, which regulates cellular energy homeostasis and reduces oxidative stress. Additionally, berberine inhibits NF-κB activation, thereby decreasing the production of pro-inflammatory cytokines [30].

Alkaloids also modulate other signaling pathways, including MAPK and PI3K/Akt, contributing to their anti-inflammatory, anti-apoptotic, and metabolic regulatory effects [31]. Furthermore, some alkaloids exhibit indirect antioxidant activity by enhancing endogenous defense systems rather than directly scavenging ROS.

The biological activity of alkaloids is highly dependent on their structural features, including the presence of heterocyclic rings and functional groups, which determine their binding affinity to molecular targets and their pharmacokinetic behavior [32].

Although alkaloids are discussed as representative mechanistic examples, the alkaloid composition of Sonoran Desert flora remains comparatively underexplored and represents an important area for future phytochemical investigation.

A comparative overview of major phytochemical classes, their signaling pathways, molecular mechanisms, and therapeutic relevance is presented in Table 1.

## 4. Molecular Mechanisms of Antioxidant Activity

**Figure 4:**
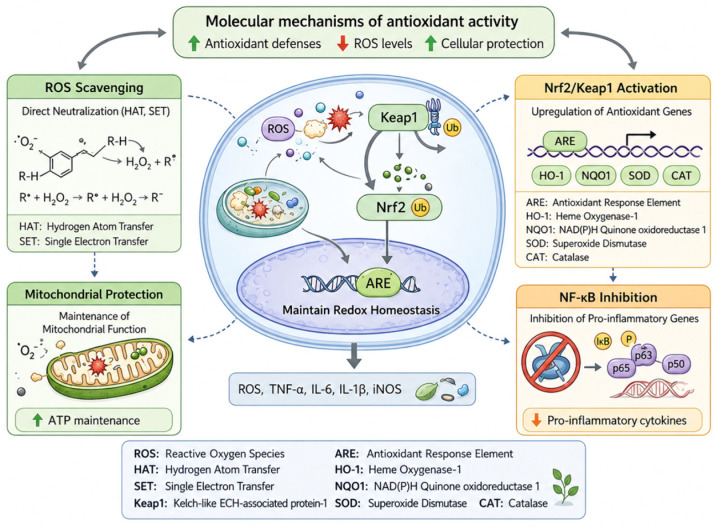
Molecular mechanisms of antioxidant activity of plant bioactives, including direct ROS scavenging, activation of Nrf2/Keap1 signaling, mitochondrial protection, and modulation of redox signaling pathways [13,14,15,16,17,18,19,20,21].

The principal antioxidant mechanisms exerted by plant bioactives, including ROS scavenging, mitochondrial protection, and Nrf2/Keap1 activation, are illustrated in Figure 4. Plant-derived bioactive compounds exert antioxidant effects through a combination of direct radical-scavenging mechanisms and indirect modulation of cellular defense systems, enabling them to regulate redox homeostasis at multiple levels. These mechanisms are particularly relevant in the context of chronic diseases, where oxidative stress contributes to cellular dysfunction, inflammation, and tissue damage [10,11]. Importantly, plant bioactives function as multi-target modulators, influencing signaling pathways, mitochondrial integrity, and gene expression, thereby providing sustained cytoprotective effects [12].

### 4.1. Direct ROS Scavenging

Plant bioactives neutralize ROS through well-established redox mechanisms, primarily HAT and SET reactions. These processes depend on the intrinsic redox potential and structural features of the compounds, particularly the presence and position of hydroxyl groups, conjugated double bonds, and aromatic systems [13,14].

In Sonoran Desert plants, chronic exposure to high solar irradiation and drought promotes the biosynthesis of highly redox-active metabolites with enhanced ROS-scavenging capacity. For instance, NDGA-rich extracts from *Larrea tridentata* and phenolic-rich extracts from *Phoradendron californicum* have demonstrated significant antioxidant activity associated with inhibition of lipid peroxidation and reduction in intracellular oxidative stress. Polyphenols and flavonoids stabilize free radicals through electron delocalization, thereby interrupting chain reactions such as lipid peroxidation. Additionally, these compounds exhibit strong metal-chelating properties, particularly toward transition metals such as Fe^2+^ and Cu^2+^, which are involved in Fenton and Haber–Weiss reactions responsible for generating highly reactive hydroxyl radicals (•OH) [15]. Recent studies have highlighted that the antioxidant efficiency of plant bioactives is influenced not only by their chemical structure but also by their interaction with cellular environments, including membrane localization and compartment-specific redox dynamics. Moreover, some compounds exhibit pro-oxidant activity under specific conditions, contributing to redox signaling and adaptive cellular responses [16].

### 4.2. Nrf2/Keap1 Pathway Activation

Nrf2 is a master regulator of antioxidant defense. Upon activation, it induces the expression of cytoprotective genes such as HO-1, NQO1, and SOD [3,11].

A central mechanism underlying the antioxidant effects of plant bioactives is the activation of the Nrf2/Keap1 signaling pathway, a master regulator of cellular redox homeostasis. Under basal conditions, Nrf2 is sequestered in the cytoplasm by Keap1 and targeted for ubiquitination and proteasomal degradation. However, oxidative stress or electrophilic phytochemicals induce conformational changes in Keap1, allowing Nrf2 stabilization and nuclear translocation [17].

Once translocated into the nucleus, Nrf2 binds to antioxidant response elements (ARE), promoting the transcription of cytoprotective genes, including:

- HO-1;
- NQO1;
- Superoxide dismutase (SOD);
- Glutathione-related enzymes (GSH, GSTs).

Plant-derived compounds such as polyphenols, flavonoids, and terpenoids act as mild electrophilic activators of Nrf2, enhancing endogenous antioxidant defenses rather than relying solely on direct ROS scavenging [18,19,20].

Importantly, Nrf2 activation is closely linked to the suppression of inflammatory pathways, particularly through negative regulation of NF-κB signaling, establishing a key redox–inflammation axis [21]. Recent studies emphasize that dysregulation of Nrf2 is associated with aging and chronic diseases, highlighting the therapeutic potential of plant bioactives as Nrf2 modulators [22].

Recent evidence suggests that phytochemicals isolated from Sonoran Desert plants may contribute to activation of endogenous antioxidant pathways, particularly Nrf2-mediated cytoprotective responses. These adaptive metabolites likely represent evolutionary responses to prolonged environmental oxidative stress conditions characteristic of arid ecosystems.

### 4.3. Mitochondrial Protection

Plant bioactives preserve mitochondrial function, reduce ROS production, and enhance mitophagy, thereby maintaining cellular energy homeostasis [15].

Mitochondria are both a primary source and target of ROS, playing a critical role in cellular redox balance. Excessive ROS production leads to mitochondrial dysfunction, characterized by loss of membrane potential, impaired ATP synthesis, and activation of apoptotic pathways [22].

Plant bioactives contribute to mitochondrial protection through multiple mechanisms:

- Preservation of mitochondrial membrane integrity;
- Reduction in mitochondrial ROS generation;
- Enhancement of mitophagy via the PINK1/Parkin pathway;
- Stimulation of mitochondrial biogenesis through PGC-1α signaling.

Polyphenols such as resveratrol and flavonoids such as quercetin have been shown to improve mitochondrial function and enhance oxidative phosphorylation efficiency, thereby restoring cellular energy metabolism [24,25].

Furthermore, plant bioactives regulate mitochondrial dynamics, including fusion and fission processes, which are essential for maintaining mitochondrial quality control. These effects contribute to reduced apoptosis and improved cellular resilience under oxidative stress conditions [26].

### 4.4. Redox Signaling Crosstalk

Moderate ROS levels regulate signaling pathways such as MAPK and PI3K/Akt. Plant bioactives restore physiological redox signaling while preventing pathological oxidative stress [16].

Reactive oxygen species are not solely harmful byproducts but also function as critical signaling molecules that regulate cellular processes such as proliferation, differentiation, and immune responses. Physiological levels of ROS modulate key signaling pathways, including:

- Mitogen-activated protein kinases (MAPK: ERK, JNK, p38);
- Phosphoinositide 3-kinase/protein kinase B (PI3K/Akt);
- AMP-activated protein kinase (AMPK).

Plant bioactives play a crucial role in maintaining redox signaling balance, preventing excessive ROS accumulation while preserving their physiological signaling functions [27].

Importantly, there is significant crosstalk between redox-sensitive pathways, particularly between Nrf2 and NF-κB, where activation of Nrf2 has been associated with suppression of inflammatory signaling. This coordinated regulation is essential for controlling oxidative stress-induced inflammation and preventing chronic disease progression [28,36].

Recent evidence also suggests that plant bioactives modulate epigenetic mechanisms, including histone modifications and microRNA expression, further influencing redox signaling and gene expression patterns [36]. These findings highlight the complexity of antioxidant mechanisms and underscore the potential of plant-derived compounds as multi-target therapeutic agents.

The ability of Sonoran Desert-derived metabolites to simultaneously modulate oxidative stress and inflammatory signaling pathways supports the concept that environmental adaptation may enhance the development of multi-target phytochemicals with therapeutic potential.

## 5. Molecular Mechanisms of Inflammation

NF-κB is a central regulator of inflammation. Its activation leads to the expression of pro-inflammatory cytokines such as TNF-α, IL-6, and IL-1β [17]. Plant bioactives inhibit NF-κB activation through multiple mechanisms, including inhibition of IKK and stabilization of IκBα [18].

Additionally, plant extracts reduce nitric oxide production in macrophages and modulate signaling pathways such as MAPK and STAT [19,20].

Inflammation is a highly coordinated biological response that involves immune cell activation, cytokine production, and complex intracellular signaling cascades. While acute inflammation is essential for host defense, chronic and dysregulated inflammation contributes to the pathogenesis of numerous diseases, including neurodegenerative disorders, metabolic syndrome, cardiovascular diseases, and cancer [17,18].

A central regulator of inflammatory responses is the NF-κB signaling pathway. Under basal conditions, NF-κB is sequestered in the cytoplasm through its interaction with the inhibitory protein IκBα. Upon stimulation by inflammatory signals such as lipopolysaccharide (LPS), ROS, or cytokines, the IκB kinase (IKK) complex phosphorylates IκBα, leading to its ubiquitination and proteasomal degradation. This process allows NF-κB (p65/p50) to translocate into the nucleus, where it promotes the transcription of pro-inflammatory genes, including tumor necrosis factor-alpha (TNF-α), interleukin-6 (IL-6), interleukin-1 beta (IL-1β), cyclooxygenase-2 (COX-2), and inducible nitric oxide synthase (iNOS) [19,20,21].

Several Sonoran Desert plant extracts have demonstrated anti-inflammatory activity associated with the suppression of NF-κB-dependent signaling pathways. These biological effects are likely linked to the elevated accumulation of stress-responsive phenolic compounds and flavonoids synthesized under arid environmental conditions.

**Figure 5:**
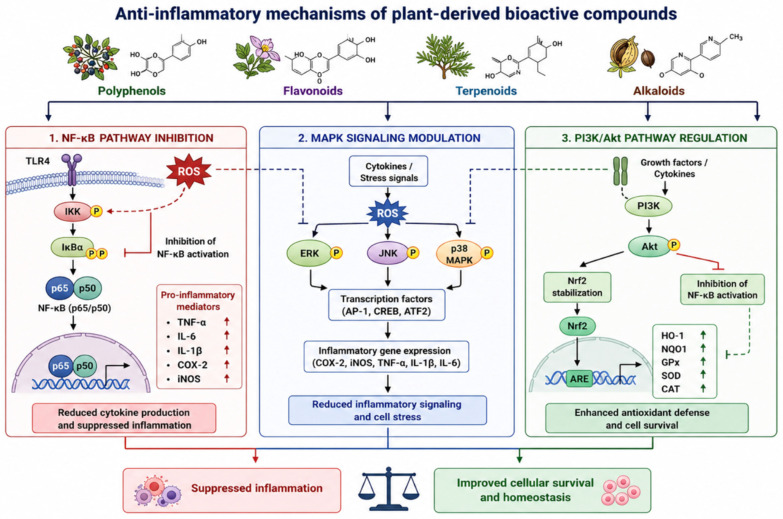
Anti-inflammatory mechanisms of plant-derived compounds. Polyphenols, flavonoids, terpenoids, and alkaloids suppress inflammatory signaling by inhibiting NF-κB activation, modulating MAPK pathways, regulating PI3K/Akt signaling, and reducing the production of pro-inflammatory mediators such as TNF-α, IL-6, IL-1β, COX-2, and iNOS [17,18,19,20,21,22,23,24,25].

The anti-inflammatory pathways targeted by plant-derived compounds, particularly NF-κB inhibition and MAPK/STAT modulation, are summarized in Figure 5.

Plant-derived bioactive compounds exert potent anti-inflammatory effects by targeting multiple steps of the NF-κB signaling cascade. These compounds inhibit NF-κB activation through mechanisms such as:

- Direct inhibition of the IKK complex;
- Stabilization of IκBα, preventing NF-κB nuclear translocation;
- Suppression of upstream signaling pathways, including Toll-like receptor 4 (TLR4) activation.

Polyphenols and flavonoids, in particular, have been shown to disrupt NF-κB signaling by modulating redox-sensitive regulatory mechanisms, thereby reducing cytokine production and inflammatory gene expression [22,23].

In addition to NF-κB inhibition, plant bioactives regulate other key inflammatory signaling pathways, including the mitogen-activated protein kinase (MAPK) pathway (ERK, JNK, p38) and the signal transducer and activator of transcription (STAT) pathway, both of which play critical roles in cytokine signaling and immune cell activation. By modulating these pathways, plant-derived compounds attenuate inflammatory responses and promote cellular homeostasis [24].

For example, extracts from *Phoradendron californicum* and *Larrea tridentata* have been associated with attenuation of inflammatory responses, reduction in oxidative damage, and modulation of cytokine production through redox-sensitive signaling mechanisms.

Another important mechanism involves the regulation of nitric oxide (NO) production in macrophages. Excessive NO generation, primarily mediated by inducible iNOS, contributes to oxidative and nitrosative stress, exacerbating tissue damage. Plant extracts have been shown to significantly reduce LPS-induced NO production in RAW 264.7 macrophages, indicating their anti-inflammatory potential [25].

These findings are particularly relevant for Sonoran Desert species, whose phytochemical profiles may provide natural multi-target modulators capable of simultaneously regulating oxidative stress and inflammatory signaling.

These coordinated mechanisms contribute to reduced oxidative stress, attenuation of chronic inflammation, and improved cellular homeostasis. Emerging evidence also highlights the role of plant bioactives in modulating the crosstalk between oxidative stress and inflammation. Activation of the Nrf2 pathway leads to suppression of NF-κB signaling, thereby linking antioxidant and anti-inflammatory responses. This coordinated regulation represents a critical mechanism through which plant-derived compounds exert protective effects in chronic diseases [26].

Furthermore, recent studies indicate that plant bioactives can influence epigenetic regulation of inflammation, including modulation of histone acetylation and microRNA expression, which further contributes to long-term anti-inflammatory effects [27].

Collectively, these findings demonstrate that plant-derived bioactive metabolites act as multi-target anti-inflammatory agents, capable of modulating key signaling pathways, reducing oxidative stress, and restoring immune homeostasis.

## 6. Experimental Approaches

The evaluation of plant-derived bioactive compounds requires a comprehensive and integrative experimental framework combining chemical, cellular, and increasingly advanced omics-based approaches to accurately characterize their antioxidant and anti-inflammatory potential. These methodologies are essential for elucidating both direct and indirect mechanisms of action, as well as for validating their translational relevance in biomedical applications [21,22].

At the chemical level, antioxidant capacity is commonly assessed using in vitro assays such as DPPH (2,2-diphenyl-1-picrylhydrazyl), ABTS (2,2′-azino-bis(3-ethylbenzothiazoline-6-sulfonic acid) and FRAP (ferric reducing antioxidant power). These assays evaluate the ability of compounds to donate electrons or hydrogen atoms and neutralize free radicals. While these methods provide rapid and reproducible measurements, they primarily reflect direct antioxidant capacity and do not fully capture biological complexity or intracellular effects [23,24].

To overcome these limitations, cell-based models are widely employed to investigate the biological activity of plant bioactives under physiologically relevant conditions. Among these, RAW 264.7 macrophages are extensively used as a model of inflammation, particularly for assessing NO production, cytokine secretion, and NF-κB activation in response to LPS stimulation. In parallel, MTT and related viability assays (e.g., resazurin, ATP-based assays) are used to evaluate cytotoxicity and cytoprotective effects, providing insight into the therapeutic window of bioactive compounds [25,26].

In addition to macrophage models, other relevant cellular systems include:

- ARPE-19 retinal pigment epithelial cells, used to study oxidative stress and retinal degeneration
- Neuronal and endothelial cell lines, for evaluating neuroprotective and vascular effects
- Hepatic and metabolic cell models, to assess systemic antioxidant responses

These models allow for the investigation of intracellular ROS production, mitochondrial function, apoptosis, and signaling pathway modulation, including Nrf2, NF-κB, MAPK, and PI3K/Akt pathways [27].

More advanced experimental approaches have emerged to provide deeper mechanistic insights. These include the following:

- Flow cytometry and fluorescence-based assays for ROS quantification (e.g., DCFH-DA);
- Western blotting and qPCR for gene and protein expression analysis;
- Mitochondrial function assays (membrane potential, oxygen consumption rate);
- High-content imaging for cellular phenotyping.

Furthermore, recent studies highlight the importance of omics technologies, including transcriptomics, proteomics, and metabolomics, to comprehensively evaluate the molecular impact of plant bioactives. These approaches enable the identification of novel targets, signaling networks, and metabolic pathways involved in antioxidant and anti-inflammatory responses [37].

Another critical aspect is the evaluation of bioavailability and pharmacokinetics, as many plant-derived compounds exhibit limited absorption, rapid metabolism, and low systemic stability. Strategies such as nanoformulations, encapsulation systems, and targeted delivery platforms are increasingly being explored to enhance the therapeutic efficacy of these compounds [38].

The most commonly used experimental methodologies for evaluating antioxidant and anti-inflammatory bioactivity are summarized in Table 2, including chemical assays, cell models, and omics-based approaches.

Despite the wide range of available methodologies, a major limitation in the field remains the lack of standardization across experimental models and assay conditions, which can affect reproducibility and comparability of results. Therefore, integrating multiple complementary approaches is essential to obtain a comprehensive understanding of the biological activity of plant bioactives and to facilitate their translation into clinical applications [43].

## 7. Sonoran Plants as a Source of Bioactives

The Sonoran Desert represents one of the most biologically diverse arid ecosystems in North America and constitutes a unique and underexplored reservoir of plant-derived bioactive compounds. Extreme environmental conditions, including intense solar radiation, prolonged drought, high temperatures, and nutrient-poor soils, impose significant physiological stress on plant species. As adaptive responses, these plants synthesize elevated levels of secondary metabolites, including polyphenols, flavonoids, terpenoids, and alkaloids, many of which exhibit potent antioxidant and anti-inflammatory bioactivity [6,7].

These stress-induced metabolic adaptations are associated with enhanced redox capacity and the ability to modulate key cellular signaling pathways involved in oxidative stress and inflammation, such as Nrf2, NF-κB, MAPK, and PI3K/Akt pathways. Consequently, plants from arid environments often display stronger bioactivity compared to species from temperate regions, highlighting their potential as sources of pharmacologically relevant compounds [8,9].

Among Sonoran plant species, *Bucida buceras* has been extensively studied for its biofunctional properties. Extracts from this species have demonstrated significant antioxidant and retinoprotective effects in retinal pigment epithelial (RPE) cells exposed to oxidative stress. These effects are associated with reduced intracellular ROS levels, improved cell viability, and modulation of antioxidant defense systems, suggesting activation of endogenous protective pathways such as Nrf2 signaling [6].

Similarly, *Phoradendron californicum* (tojis), a hemiparasitic plant native to the Sonoran Desert, has been reported to exhibit high phenolic content and strong antioxidant capacity. Experimental studies indicate that extracts from this species can attenuate oxidative damage and reduce inflammatory responses, potentially through inhibition of NF-κB activation and modulation of cytokine production [7].

Among additional Sonoran Desert species, *Larrea tridentata* has attracted considerable attention due to its high content of NDGA, lignans, and flavonoid-derived compounds. NDGA is recognized for its potent antioxidant activity, including ROS scavenging, inhibition of lipid peroxidation, and modulation of redox-sensitive signaling pathways such as NF-κB and Nrf2. Several studies have also associated *Larrea tridentata* extracts with anti-inflammatory, neuroprotective, and anticancer properties [48].

Similarly, *Opuntia ficus-indica* contains a diverse phytochemical profile rich in betalains, flavonoids, phenolic acids, vitamin C, and carotenoids. These metabolites contribute to antioxidant defense, mitochondrial protection, and regulation of inflammatory mediators. Experimental evidence suggests that *Opuntia* extracts may improve metabolic homeostasis and attenuate oxidative stress-related damage [49].

Another relevant species is *Agave deserti*, which produces saponins, flavonoids, and fructan-associated metabolites with reported antioxidant and immunomodulatory activities. Adaptation to arid environmental conditions, including prolonged drought and high ultraviolet exposure, likely promotes the accumulation of stress-responsive secondary metabolites with enhanced biological activity [50].

In addition to these species, other Sonoran plants including cacti, shrubs, and desert-adapted herbs have shown promising bioactivity, although many remain insufficiently characterized at the molecular level. The diversity of phytochemicals present in these plants suggests a wide range of biological activities, including cytoprotective, anti-inflammatory, neuroprotective, and metabolic regulatory effects [10].

Despite these promising findings, several limitations hinder the full exploitation of Sonoran plant bioactives. These include the following:

- Limited phytochemical characterization at the molecular level;
- Lack of standardized extraction and analytical protocols;
- Insufficient mechanistic studies linking bioactivity to specific signaling pathways;
- Scarcity of in vivo and clinical studies.

Recent advances in analytical techniques, such as mass spectrometry-based metabolomics, high-performance liquid chromatography (HPLC), and nuclear magnetic resonance (NMR), provide powerful tools for the identification and characterization of bioactive compounds. Furthermore, integration with omics approaches and systems biology enables a more comprehensive understanding of their mechanisms of action [11,12].

Importantly, the exploration of Sonoran plant bioactives aligns with current trends in bioprospecting and sustainable drug discovery, emphasizing the valorization of regional biodiversity and the development of nutraceuticals and pharmacological agents derived from natural sources [13].

Future research should prioritize:

- Detailed phytochemical profiling and structure–activity relationships;
- Mechanistic studies focusing on redox and inflammatory signaling pathways;
- Development of delivery systems to improve bioavailability;
- Translation of in vitro findings into in vivo and clinical studies.

Collectively, Sonoran plants represent a promising and largely untapped source of bioactive compounds with significant potential for the prevention and treatment of oxidative-stress-related diseases.

## 8. Therapeutic Applications

The therapeutic relevance of plant-derived bioactives across multiple chronic diseases and associated signaling pathways is summarized in Table 3. Plant-derived bioactive compounds have emerged as promising therapeutic agents due to their ability to modulate multiple molecular pathways involved in oxidative stress, inflammation, and cellular dysfunction. Their multi-target and pleiotropic effects make them particularly attractive for the prevention and treatment of complex diseases, where single-target therapies often fail to achieve sustained efficacy [22].

### 8.1. Neurodegenerative Diseases

In neurodegenerative disorders such as Alzheimer’s disease, Parkinson’s disease, and age-related macular degeneration (AMD), oxidative stress and chronic inflammation play central roles in neuronal damage and disease progression. Plant bioactives, particularly polyphenols and flavonoids, have demonstrated neuroprotective effects through the activation of Nrf2 signaling, inhibition of NF-κB, and reduction in mitochondrial dysfunction [22].

Compounds such as quercetin, resveratrol, and catechins have been shown to reduce neuronal apoptosis, improve synaptic function, and attenuate neuroinflammation. Additionally, their ability to modulate microglial activation and reduce ROS production highlights their potential in preventing neurodegenerative processes [25,26].

### 8.2. Cardiovascular Diseases

Oxidative stress and endothelial dysfunction are key contributors to cardiovascular diseases, including atherosclerosis and hypertension. Plant bioactives exert cardioprotective effects by improving endothelial function, reducing oxidative damage, and inhibiting inflammatory pathways [25].

Polyphenols have been shown to enhance NO bioavailability, improve vascular relaxation, and reduce lipid peroxidation. Furthermore, their ability to inhibit NF-κB signaling and modulate lipid metabolism contributes to reduced plaque formation and vascular inflammation [22,25].

### 8.3. Metabolic Disorders

Metabolic diseases such as diabetes and obesity are characterized by chronic low-grade inflammation, insulin resistance, and oxidative stress. Plant-derived compounds, including flavonoids and alkaloids such as berberine, have demonstrated significant potential in regulating metabolic pathways.

These compounds activate AMPK signaling, improve glucose uptake, and reduce inflammatory responses, thereby enhancing insulin sensitivity and metabolic homeostasis. Additionally, their antioxidant properties contribute to the prevention of diabetic complications, including vascular and retinal damage [14,30].

### 8.4. Cancer Prevention and Therapy

**Figure 6:**
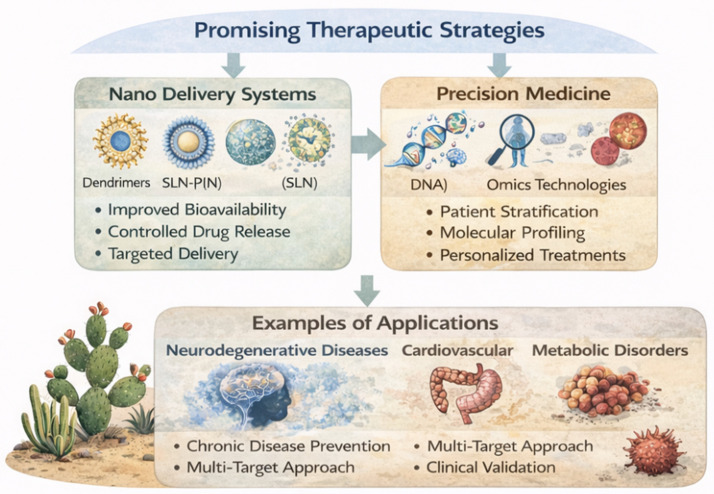
Therapeutic applications of plant-derived bioactives in major chronic diseases, including neurodegenerative, cardiovascular, metabolic, and cancer-related conditions [21,22,23,24,25,26,27,28,29,30].

Chronic oxidative stress and inflammation are closely linked to cancer initiation and progression. Plant bioactives have shown potential for cancer prevention and therapy through their ability to modulate multiple hallmarks of cancer, including proliferation, apoptosis, angiogenesis, and metastasis [30]. The broad therapeutic applications of plant bioactives, including neurodegenerative, cardiovascular, metabolic, and oncological disorders, are schematically illustrated in Figure 6.

Polyphenols and terpenoids have been reported to induce apoptosis in cancer cells, inhibit tumor growth, and reduce oxidative DNA damage. Moreover, these compounds can modulate signaling pathways such as PI3K/Akt, MAPK, and NF-κB, thereby suppressing tumors, promoting inflammation, and enhancing cellular defense mechanisms [51].

### 8.5. Translational and Nutraceutical Potential

The therapeutic potential of plant bioactives extends beyond pharmacological applications to include their use as nutraceuticals and functional food components. Their safety profile, natural origin, and broad-spectrum activity make them attractive candidates for long-term disease prevention strategies [52].

However, several challenges remain, including:

Low bioavailability and rapid metabolism;

Variability in plant composition;

Limited clinical evidence.

To address these limitations, recent studies have focused on nanotechnology-based delivery systems, such as nanoparticles, liposomes, and nanoemulsions, which enhance stability, absorption, and targeted delivery of bioactive compounds [53].

### 8.6. Future Clinical Perspectives

Despite promising preclinical evidence, the translation of plant bioactives into clinical applications requires:

Well-designed clinical trials;

Standardization of extracts;

Identification of optimal dosing strategies.

Integration of precision medicine approaches and biomarker-guided therapies may further enhance the therapeutic potential of these compounds in personalized healthcare [54].

**Figure 7:**
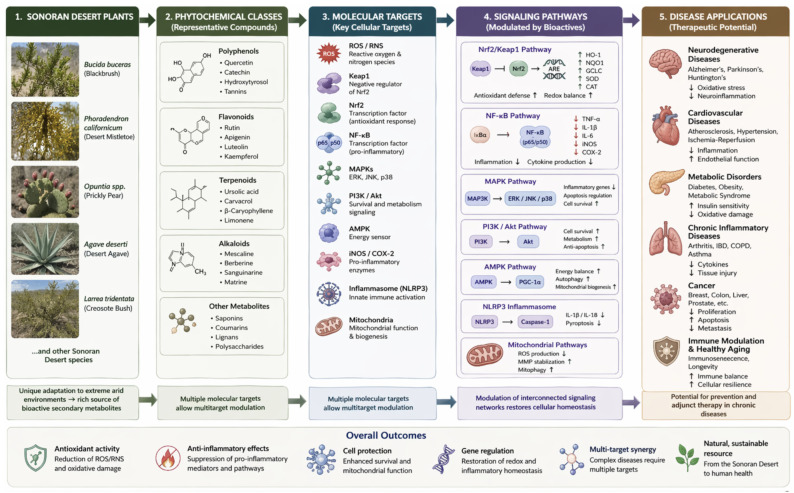
Integrative overview of Sonoran Desert plant bioactives: from natural sources to molecular mechanisms and therapeutic applications.

To provide a systems-level perspective, Figure 7 integrates the progression from Sonoran Desert plant sources and phytochemical classes to molecular targets, signaling pathways, and therapeutic applications. This integrative framework highlights the multi-target nature of plant-derived metabolites and their potential relevance in chronic disease prevention and precision medicine.

## 9. Limitations and Future Perspectives

Despite promising results, several limitations remain, including low bioavailability, variability in plant composition, and a lack of clinical studies. Future research should focus on nanodelivery systems, pharmacokinetics, and clinical validation [55].

Despite the growing body of evidence supporting the antioxidant and anti-inflammatory potential of plant-derived bioactive compounds, several critical limitations hinder their translation into clinical and therapeutic applications. One of the major challenges is their low bioavailability, which is influenced by poor solubility, limited intestinal absorption, rapid metabolism, and systemic elimination. Many polyphenols and flavonoids undergo extensive biotransformation in the liver and gut microbiota, resulting in reduced concentrations of active metabolites at target tissues [56,57].

Another important limitation is the variability in phytochemical composition, which depends on multiple factors including plant species, geographical origin, environmental conditions, harvesting time, and extraction methods. This variability complicates the reproducibility of experimental results and the standardization of plant extracts, which is essential for clinical application and regulatory approval [58].

Furthermore, most studies investigating plant bioactives are based on in vitro models and preclinical data, with limited validation in animal models and human clinical trials. This gap between experimental findings and clinical evidence represents a significant barrier to the development of plant-derived therapeutics. Additionally, the lack of standardized experimental protocols and dosing regimens further complicates the interpretation and comparison of results across studies [59].

Another emerging limitation is related to the complex interactions between bioactive compounds and biological systems, including their modulation by the gut microbiota. Recent studies suggest that the biological activity of plant metabolites may depend not only on their native structure but also on their microbial-derived metabolites, which can have distinct pharmacological effects [60,61].

To overcome these challenges, future research should focus on the development of advanced delivery systems, including nanoparticles, liposomes, polymeric carriers, and nanoemulsions. These nanotechnology-based approaches can improve the stability, bioavailability, and targeted delivery of plant bioactives, enhancing their therapeutic efficacy [57,62].

In addition, integrating pharmacokinetic and pharmacodynamic studies is essential to better understand absorption, distribution, metabolism, and excretion (ADME) profiles. This information is critical for optimizing dosing strategies and ensuring safety and efficacy in clinical applications [63].

Recent advances in omics technologies, such as transcriptomics, proteomics, and metabolomics, offer powerful tools for elucidating the molecular mechanisms of plant bioactives at a systems level. These approaches enable the identification of novel biomarkers and signaling networks, facilitating the development of precision medicine strategies [57,62].

Moreover, future studies should prioritize:

Well-designed randomized clinical trials;

Standardization of plant extracts and bioactive compounds;

Elucidation of structure–activity relationships;

Exploration of synergistic effects between multiple compounds.

Finally, the integration of plant bioactives into personalized and precision medicine frameworks represents a promising direction for future research. By combining molecular profiling, biomarker identification, and targeted therapeutic strategies, plant-derived compounds could play a significant role in the prevention and treatment of chronic diseases [30].

## 10. Conclusions

Plant-derived bioactive metabolites from arid environments represent a promising strategy for modulating oxidative stress and inflammation through multi-target mechanisms involving key pathways such as Nrf2, NF-κB, MAPK, and PI3K/Akt. These compounds exhibit significant antioxidant, anti-inflammatory, and cytoprotective effects, supporting their relevance in the prevention and management of chronic diseases.

Sonoran Desert plants constitute an underexplored source of bioactive molecules with enhanced functional properties driven by environmental stress adaptation. However, limitations related to bioavailability, variability, and lack of clinical validation remain critical challenges. Future research should focus on mechanistic validation, standardized formulations, and translational approaches, including nanodelivery systems and clinical studies, to fully exploit the therapeutic potential of these compounds. Collectively, Sonoran Desert-derived phytochemicals represent a promising frontier for multi-target molecular therapeutics, bridging biodiversity-driven discovery with precision medicine approaches.

## Figures and Tables

**Table 1:** Major classes of plant-derived bioactive metabolites: molecular mechanisms, signaling pathways, and therapeutic relevance.

Class	Representative Compounds	Sonoran Sources	Main Mechanisms	Key Pathways	Biological Effects	Therapeutic Relevance	References
Polyphenols	Resveratrol, gallic acid, curcumin, NDGA	*Bucida buceras*, *Larrea tridentata*, *Phoradendron californicum*	ROS scavenging, metal-chelation, antioxidant activation	Nrf2/Keap1, NF-κB, MAPK	Antioxidant, anti-inflammatory, cytoprotective	Neurodegeneration, cardiovascular diseases, and cancer	[3,5,11,15,16,17,18]
Flavonoids	Quercetin, catechin, kaempferol, rutin	*Opuntia ficus-indica*, *Sonoran cacti*	ROS neutralization, cytokine suppression, and IκBα stabilization	NF-κB, MAPK, PI3K/Akt, AMPK	Anti-inflammatory, neuroprotective, cardioprotective	Diabetes, metabolic syndrome, atherosclerosis	[3,12,16,19,20,21,22,23,24]
Terpenoids	β-carotene, lycopene, limonene	*Agave deserti* and Sonoran flora	Lipid peroxidation inhibition, mitochondrial protection	PI3K/Akt, Nrf2	Cytoprotective, antioxidant, and metabolic regulation	Cardiovascular diseases, chronic inflammation, and cancer	[13,15,25,26,27,28]
Alkaloids	Berberine, caffeine, quinine	Desert alkaloid-containing plants	AMPK activation, indirect antioxidant activity	AMPK, NF-κB, MAPK	Anti-inflammatory, cytoprotective	Diabetes, inflammatory diseases, and cancer	[14,18,30,31,33,34,35]

Abbreviations: ROS, reactive oxygen species; NDGA, nordihydroguaiaretic acid; Nrf2, nuclear factor erythroid 2-related factor 2; NF-κB, nuclear factor kappa B; MAPK, mitogen-activated protein kinase; PI3K/Akt, phosphoinositide 3-kinase/protein kinase B; AMPK, AMP-activated protein kinase.

**Table 2:** Experimental approaches for evaluating antioxidant and anti-inflammatory bioactivity of plant-derived bioactives.

Method	Experimental Model	Purpose	Measured Parameters	Advantages	Limitations	References
**DPPH assay**	Chemical (in vitro)	Evaluate radical scavenging capacity	IC50, % inhibition	Simple, rapid, reproducible	Non-physiological	[39,40]
**ABTS assay**	Chemical (in vitro)	Measure antioxidant capacity	TEAC values	Applicable to hydrophilic and lipophilic compounds	Limited biological relevance	[41,42]
**FRAP assay**	Chemical (in vitro)	Assess reducing power	Fe^2+^ reduction capacity	Fast and inexpensive	Does not reflect ROS scavenging in cells	[41,43]
**MTT/viability assays**	Cell lines (RAW 264.7, ARPE-19)	Assess cytotoxicity and cytoprotection	Cell viability (%)	Widely used, quantitative	Indirect measurement of viability	[44]
**DCFH-DA assay**	Cellular models	Measure intracellular ROS levels	Fluorescence intensity	Reflects intracellular oxidative stress	Probe instability, non-specificity	[43,45]
**NO production assay**	RAW 264.7 macrophages	Evaluate anti-inflammatory bioactivity activity	Nitrite levels (Griess assay)	Relevant inflammation model	Limited to macrophage response	[43,44]
**Western blot/qPCR**	Cellular models	Analyze signaling pathways	Protein/gene expression (Nrf2, NF-κB, MAPK)	Mechanistic insight	Time-consuming, semi-quantitative	[46,47]
**Mitochondrial assays**	Cellular models	Assess mitochondrial function	ΔΨm, ATP production	Functional relevance	Requires specialized equipment	[45]
**Omics approaches**	Systems biology	Identify molecular targets	Transcriptomics, proteomics, metabolomics	Comprehensive analysis	High cost, complex interpretation	[43,46]
**Nanodelivery evaluation**	In vitro/in vivo	Improve bioavailability	Improve bioavailability	Enhances therapeutic potential	Limited clinical validation	[43]

**Table 3:** Therapeutic applications of plant-derived bioactive metabolites: molecular targets, signaling pathways, and clinical relevance.

Disease	Pathophysiological Mechanisms	Representative Compounds	Molecular Mechanisms	Key Signaling Pathways	Therapeutic Effects	Clinical Relevance	References
**Neurodegenerative** diseases (AD, PD, AMD)	Oxidative stress, neuroinflammation, mitochondrial dysfunction	Quercetin, resveratrol, catechins	ROS reduction, inhibition of microglial activation, mitochondrial protection	Nrf2/Keap1, NF-κB inhibition, MAPK modulation	Neuroprotection, reduced apoptosis, improved synaptic function	Potential prevention and progression delay	[3,5,11,22]
**Cardiovascular** diseases	Endothelial dysfunction, oxidative stress, and inflammation	Polyphenols, flavonoids	Increased NO bioavailability, reduced lipid peroxidation	PI3K/Akt, NF-κB inhibition	Improved vascular function, anti-atherogenic effects	Cardioprotection, reduced risk factors	[5,22,25]
**Metabolic disorders** (diabetes, obesity)	Insulin resistance, chronic inflammation, oxidative stress	Berberine, flavonoids	AMPK activation, improved glucose metabolism, and anti-inflammatory effects	AMPK, NF-κB inhibition PI3K/Akt	Improved insulin sensitivity, reduced inflammation	Glycemic control, metabolic regulation	[14,30]
**Cancer**	DNA damage, chronic inflammation, and uncontrolled proliferation	Polyphenols, terpenoids	Induction of apoptosis, inhibition of proliferation, and ROS modulation	PI3K/Akt, MAPK, NF-κB inhibition	Anti-proliferative, anti-angiogenic effects	Cancer prevention and adjunct therapy	[3,26,27]
**Inflammatory diseases**	Cytokine overproduction, immune dysregulation	Flavonoids, alkaloids	Inhibition of cytokine production, suppression of inflammatory mediators	NF-κB, MAPK, STAT pathways	Reduced inflammation, immune modulation	Chronic disease management	[18,19,20,25]

## Data Availability

The original contributions presented in this study are included in the article. Further inquiries can be directed to the corresponding author.
